# Histopathological Advantages of En Bloc Resection in Non–muscle-invasive Bladder Cancer: A Multinational Randomised Controlled Clinical Trial

**DOI:** 10.1016/j.euros.2026.06.005

**Published:** 2026-07-02

**Authors:** Ninna K. Nielsen, Rikke V. Milling, Peter B. Hjort, Juan L. Vásquez, Erik S. Haug, Frederikke E. Sørensen, Kasper Ø. Olsen, Knud Fabrin, Egils Vjaters, Trine M. Rudlang, Teesi Sepp, Pertti Nurminen, Louise Geertsen, Gitte W. Lam, Jakob K. Jakobsen, Charlotte Graugaard-Jensen, Sanja Stifter-Vretenar, Jørgen B. Jensen, Pernille S. Kingo

**Affiliations:** a Department of Urology, Aarhus University Hospital, Aarhus, Denmark; b Department of Clinical Medicine, Aarhus University, Aarhus, Denmark; c Department of Urology, Gødstrup Regional Hospital, Gødstrup, Denmark; d Department of Urology, Copenhagen University Hospital – Zealand University Hospital Roskilde, Roskilde, Denmark; e Department of Clinical Medicine, University of Copenhagen, Copenhagen, Denmark; f Department of Urology, Vestfold Hospital Trust, Tønsberg, Norway; g Institute of Cancer Genomics and Informatics, Oslo University Hospital, Oslo, Norway; h Institute of Clinical Medicine, University of Bergen, Bergen, Norway; i Department of Urology, Sygehus Lillebælt, Vejle, Denmark; j Department of Urology, Aalborg University Hospital, Aalborg, Denmark; k Urological Center, Paula Stradina Clinical University Hospital, Riga, Latvia; l Department of Urology, Herlev and Gentofte Hospital, Herlev, Denmark; m General and Oncology Urology Centre, North Estonia Medical Centre, Tallinn, Estonia; n Department of Urology, Turku University Hospital, Turku, Finland; o Department of Urology, Odense University Hospital, Odense, Denmark; p Department of Pathology, Aarhus University Hospital, Aarhus, Denmark; q Greenland Centre for Health Research, Institute for Health and Nature, Ilisimatusarfik/University of Greenland, Nuuk, Greenland

**Keywords:** Bladder cancer, En bloc resection, Non–muscle-invasive bladder cancer, Pathological specimen quality, Pathology review, Randomised controlled clinical trial

## Abstract

**Background and objective:**

En bloc resection of bladder tumours (ERBT) has been proposed as an alternative to conventional transurethral resection of bladder tumours (cTURBT) for non–muscle-invasive bladder cancer (NMIBC), potentially improving clinical outcomes and pathological specimen quality. This randomised controlled trial (RCT) compared pathological staging and grading consistency, thermal damage, and specimen orientation between ERBT and cTURBT.

**Methods:**

In this multinational RCT, patients with suspected NMIBC tumours measuring 1–4 cm were randomised 1:1 to ERBT or cTURBT. A central pathology review was conducted. The primary end point was T-stage concordance between central review and initial pathology assessment. Secondary outcomes included thermal damage and loss of orientation (fragments lacking epithelial cells or detrusor muscle). Surgical outcomes and complications were also evaluated.

**Key findings and limitations:**

Across 11 sites in five countries, 220 patients were randomised (110 ERBT, 110 cTURBT). For pathology review, 209 cases (103 ERBT, 106 cTURBT) were available. T-stage concordance was 80% (ERBT) versus 81% (cTURBT), corresponding to a difference of −1.5% (95% confidence interval [CI], −12 to 9.3; *p* = 0.8). Fragments with loss of orientation were 26% less frequent with ERBT (95% CI, −40 to −13; *p* < 0.001). Median thermal damage was 5.0% lower with ERBT (95% CI, −9.0 to −1.0; *p* < 0.001). The main limitation was the lack of standardised specimen-handling procedures across sites, leading to suboptimal preparation of some ERBT specimens.

**Conclusions and clinical implications:**

T-stage concordance was similar between the ERBT and cTURBT groups. ERBT specimens showed improved orientation and reduced thermal damage. Standardised specimen-handling protocols are essential to fully realise the pathological advantages of ERBT.

## Take Home Message

In this randomised trial, en bloc resection did not improve pathological T-stage concordance compared with conventional transurethral resection of bladder tumours. En bloc resection of bladder tumour specimens showed improved orientation and less thermal damage. Supoptimal specimen handling underscored the need for standardised specimen-handling protocols.

ADVANCING PRACTICE **What does this study add?**

This study provides randomised evidence on the pathological effects of en bloc resection compared with conventional transurethral resection of bladder tumours in non–muscle-invasive bladder cancer. Although En bloc resection of bladder tumours (ERBT) did not improve pathological staging or grading concordance, it resulted in better specimen orientation, fewer nondiagnostic fragments, and less thermal damage. These findings indicate that ERBT can yield higher-quality specimens when handled appropriately and underscore the importance of standardised specimen-handling protocols.

**Clinical Relevance**

This multinational randomised clinical trial demonstrates that en bloc resection for non–muscle-invasive bladder cancer improves histopathological specimen quality by reducing thermal artefact and preserving tissue orientation, although it did not improve T-stage concordance compared with conventional TURBT. The findings highlight the potential of ERBT to facilitate more reliable pathological assessment and T1 substaging, while underscoring the critical importance of surgeon expertise and standardised specimen-handling protocols for optimal implementation in clinical practice. Associate Editor: M. Carmen Mir, M.D; PhD.

**Patient Summary**

We studied two surgical techniques for removing superficial bladder tumours. Removing tumours in one piece did not improve cancer staging compared with standard surgery, but produced tissue samples with improved orientation and less thermal damage.

## 1 Introduction

Bladder cancer is a common malignancy with >600,000 cases diagnosed annually worldwide [1]. Although muscle-invasive bladder cancer (MIBC) is an aggressive disease requiring major surgery associated with substantial morbidity, non-MIBC (NMIBC) is associated with lower mortality but higher recurrence risk. Consequently, patients with NMIBC require intensive follow-up, imposing a considerable burden on both patients and health care systems [2].

The cornerstone of NMIBC diagnosis and management is transurethral resection of bladder tumours (TURBT). In this procedure, tumours are endoscopically resected and retrieved for histopathological assessment to determine tumour stage (T-stage) and grade, which guide subsequent treatment decisions. However, conventional TURBT (cTURBT) fragments the tumour before removal, leading to two problems [3]. Incomplete resection and potential tumour cell scattering may increase the risk of recurrence, and pathological evaluation is compromised because tumour orientation is lost and surgical margins cannot be assessed [4]. Therefore, infiltration into the suburothelial tissue or detrusor muscle (DM) may be underestimated.

En bloc resection of bladder tumours (ERBT) has been proposed as an alternative to cTURBT. In ERBT, the tumour is removed in one piece, preserving its architecture and potentially ensuring free surgical margins. The method was first described in 1997 [5], and subsequent studies have demonstrated its feasibility and safety [6], [7]. More recent trials have reported improved histopathological specimen quality and favourable oncological outcomes with ERBT [8], [9].

The presence of DM has been used as a surrogate marker for specimen quality [10]. However, DM may also be present in suboptimal specimens, limiting discriminatory value and underscoring the need for more comprehensive measures of specimen quality.

Improving specimen quality in NMIBC could enhance diagnostic accuracy, enable more reliable substaging, and potentially reduce the need for repeat resections (reTURBTs), benefitting both patients, clinicians, and the health care system. Given these potential advantages, it is essential to determine whether ERBT provides superior pathological specimens compared with cTURBT.

In this RCT with central pathology review, the primary end point was the proportion of specimens with unchanged T-stage following central pathology review, potential reTURBT, and cystectomy. We hypothesised that ERBT would yield a higher concordance than cTURBT. The trial aimed to evaluate whether ERBT offers histopathological advantages over cTURBT in NMIBC with emphasis on staging and grading consistency and specimen orientation.

## 2 Methods

### 2.1 Study design

This multinational, open-label, RCT was conducted from March 2022 to September 2024. In total, 220 patients were enrolled across 11 sites in Denmark (7 sites), Norway, Finland, Latvia, and Estonia. Patients were randomised 1:1 to cTURBT or ERBT, stratified by sex and recruitment site, using block randomisation with varying block sizes (4/6/8) generated by an independent external data manager. Randomisation was conducted in Research Electronic Data Capture (REDCap), a secure, web-based software platform [11]. Written informed consent was obtained before randomisation.

### 2.2 Eligibility criteria

Patients aged ≥18 yr, able to provide informed consent, with a primary papillary bladder tumour of 1–4 cm were eligible. Initially, inclusion of tumours up to 6 cm was planned, but the upper limit was reduced due to extraction limitations with the available equipment.

Solid-appearing tumours and flat lesions were not eligible. Patients with multiple tumours or concomitant flat lesions were eligible if one tumour met the size and morphology criteria. Lesions after ≥5 yr recurrence-free were considered primary.

Exclusion criteria: suspected muscle-invasive disease (by cystoscopy or imaging), tumours in a diverticulum, or if ERBT was deemed technically infeasible at initial cystoscopy. Tumour size was assessed on imaging when available, otherwise during cystoscopy.

### 2.3 Surgical technique and specimen handling

Patients with cTURBT were managed according to local standards. Patients with ERBT were booked with surgeons with experience in the ERBT technique. The local Principal Investigator (PI) was responsible for appointing ERBT surgeons; no formal quality assessment was conducted.

#### 2.3.1 cTURBT

cTURBT was performed under general or spinal anaesthesia, according to local practice. Tumours were resected piecemeal with a bipolar wire loop.

#### 2.3.2 ERBT

ERBT was also performed under general or spinal anaesthesia. A bipolar wire loop, a bipolar J-shaped electrode, or a laser was used (surgeon’s preference). The ERBT technique followed previously described methods [12], [13]. Tumour margin was outlined with a 5-mm margin of normal mucosa, and the detrusor layer was reached using an intermittent burst cutting technique. For specimen retrieval, PolyCatch endobags (Medi-Globe GmbH, Rohrdorf-Achenmühle, Germany) were provided; alternative retrieval methods included other endobags, snares, biopsy forceps, an Ellik evacuator, or water flow through the resectoscope. Modified ERBT—segmenting the specimen during or after resection—was permitted when extraction was otherwise infeasible.

#### 2.3.3 Postoperative management

Postoperative management (routine catheterisation, bladder irrigation, and single-instillation intravesical chemotherapy) followed local protocols.

#### 2.3.4 Specimen handling

Surgeons were requested to ink the resection base of ERBT specimens after extraction, while specimen orientation was otherwise according to local practice. ERBT specimens were intended to be sliced perpendicular to the resection base.

### 2.4 Pathology review

Pathology review was performed by the same specialised uropathological consultant (SSV) at the primary investigation centre. In 30 cases where SSV performed the initial assessment, the review was performed by a second uropathological consultant. These two uropathologists agreed in advance on the specific parameters to be assessed at revision and the criteria for their evaluation.

Specimens were submitted for review as physical slides or digital scans.

T1 substaging is routinely performed in Denmark; for sites without initial substaging, any pT1 substage at review was considered concordant with an initial pT1 diagnosis.

### 2.5 Outcome measures and data management

The primary outcome was T-stage concordance between the initial pathological assessment and findings at central pathology review, and at potential reTURBT and cystectomy.

Secondary outcomes at pathology review included unchanged grade, thermal damage, and loss of specimen orientation (fragments lacking epithelial cells or DM).

Surgical outcomes included surgeon expertise (resident, urologist with <5, or >5 yr of experience), operative time, peri- and postoperative complications, and, for patients undergoing ERBT, successful tumour resection and extraction.

Data were collected by the national PI and managed in REDCap [11].

### 2.6 Sample size calculation

Based on retrospective data on residual tumour at reTURBT and cystectomy, we expected 75% of patients to have an unchanged T-stage after cTURBT [14]. For the ERBT group, we estimated 90% T-stage concordance, anticipating a reduction in upstaging from approximately 25% to 10%. This was based on data reporting ∼20% upstaging of T1 tumours at reTURBT with conventional resection [15], and our previously reported ∼15% rate of T-stage discordance at pathological revision alone [14].

Using a two-sided α of 0.05 and a power (1–β) of 0.80, 100 patients per group were required. Assuming a 10% dropout rate, the target sample size was 220 patients (110 per group).

### 2.7 Statistical analyses

Categorical variables are presented as frequencies and percentages, and continuous variables as medians with first and third quartiles. Missing values are displayed as “unknown”.

Comparisons between groups were performed using Fisher’s exact or Pearson‘s chi-squared test for categorical variables and the Wilcoxon rank sum test for continuous variables.

An analysis of covariance (ANCOVA) was performed to adjust for surgeon expertise, with thermal damage as the outcome, randomisation group as the predictor, and surgeon as a three-level categorical covariate. The presence of fragments with loss of orientation was compared using logistic regression for the adjusted analysis with surgeon expertise as a covariate.

ANCOVA assumptions were evaluated using residuals versus fitted plots and a Q-Q plot. Logistic regression assumptions were evaluated by inspection of contingency tables for complete separation and Cook’s distance for influential observations.

The primary analyses followed the intention-to-treat principle. A per-protocol (PP) analysis was performed, including only ERBT specimens that resembled ERBT morphology at pathology review. ERBT cases that did not meet these criteria were analysed within the cTURBT PP group.

Statistical analyses were performed using R statistical software, version 4.3.0 (2023-04-21) [16].

### 2.8 Ethical approval

The study was approved by the National Ethics Committees in participating countries. The primary protocol was approved in Denmark by the Regional Committee on Health Research Ethics for the Central Denmark Region (1-10-72-31-21) and the Central Denmark Region research registry (1-16-02-25-21), approved by the Danish Data Protection Agency. The study was registered on clinicaltrials.gov (NCT05223491).

## 3 Results

### 3.1 Patients and procedures

**Fig. 1:**
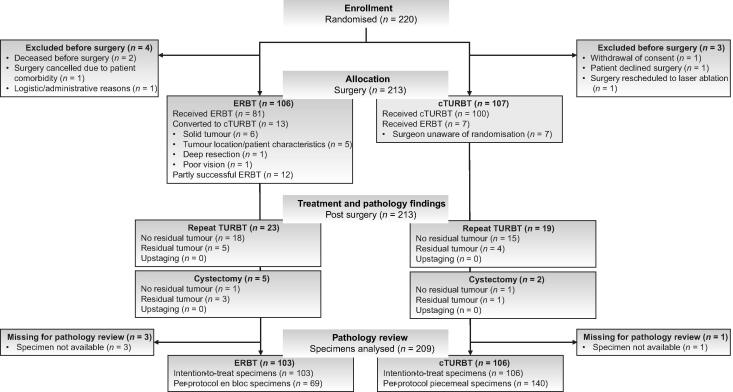
CONSORT flow diagram illustrating patient enrolment, surgical allocation, initial treatment, and inclusion in the pathology review analyses.

From March 2022 to September 2024, 220 patients were enrolled (110 cTURBT, 110 ERBT). Seven patients were excluded before surgery (Fig. 1). Patient characteristics were similar in the two groups (Table 1).

**Table 1:** Patient characteristics at enrolment

	**Randomisation**	
	**En bloc**, *n* = 110	**cTURBT**, *n* = 110
**Country, *n* (%)**		
Denmark	90 (82%)	89 (81%)
Norway	11 (10%)	12 (11%)
Estonia	2 (1.8%)	3 (2.7%)
Latvia	4 (3.6%)	4 (3.6%)
Finland	3 (2.7%)	2 (1.8%)
**No. of men, *n* (%)**	78 (71%)	75 (68%)
**Patient age, median (Q1–Q3)**	71 (63–76)	71 (65–76)
**BMI, median (Q1–Q3)**	26.5 (24.0–30.0)	28.0 (25.0–30.0)
Unknown	2	3
**Smoking status, *n* (%)**		
Never smoker	13 (12%)	18 (16%)
Former smoker	51 (46%)	47 (43%)
Current smoker	29 (26%)	28 (25%)
Unknown	17 (15%)	17 (15%)
**ASA Classification, *n* (%)**		
1	25 (23%)	25 (23%)
2	57 (52%)	59 (54%)
3	25 (23%)	26 (24%)
4	2 (1.8%)	0 (0%)
Unknown	1	0
**Tumour diameter, mm, median (Q1**–**Q3)**	20 (15–30)	20 (15–25)
Unknown	0	2
**Number of tumours, *n* (%)**		
1	75 (71%)	73 (68%)
2–4	30 (28%)	31 (29%)
4–7	1 (0.9%)	2 (1.9%)
>7	0 (0%)	1 (0.9%)

ASA = American Society of Anesthesiologists; BMI = body mass index; cTURBT = conventional transurethral resection of bladder tumour.

ERBT was successful in 81 cases (76%), partly successful (modified ERBT) in 12 cases (11%), and converted to cTURBT in 13 cases (12%; Fig. 1). ERBT was performed by more experienced surgeons (Table 2) and had a longer median operative time than cTURBT: 38 min (Q1, Q3: 25, 58) versus 27 min (Q1, Q3: 20, 41). Distributions of T-stage, grade, and EORTC risk groups were similar in the two groups. DM was absent in 38% of ERBT and 35% of cTURBT specimens.

**Table 2:** Surgical characteristics and initial pathology

	**Randomisation**	
	**En bloc**, *n* = 106	**cTURBT**, *n* = 107
**Resection of the tumour en bloc, *n* (%)**		
Successful	81 (76%)	
Partly successful/modified ERBT	12 (11%)	
Converted to cTURBT	13 (12%)	
**Extraction of the tumour en bloc, *n* (%)**^a^	52 (64%)	
**Surgeon's expertise**		
Resident	16 (15%)	50 (47%)
Urologist < 5 yr experience	51 (48%)	24 (22%)
Urologist ≥ 5 yr experience	34 (32%)	29 (27%)
Unknown	5 (4.7%)	4 (3.7%)
**Duration of surgery, median (Q1**–**Q3)**	38 (25–58)	27 (20–41)
**T-stage, *n* (%)**		
pTa	76 (72%)	80 (75%)
Could not be specified pTa/pT1	1 (0.9%)	4 (3.8%)
pT1a	16 (15%)	10 (9.4%)
pT1b	3 (2.8%)	1 (0.9%)
pT1 unspecified	4 (3.8%)	5 (4.7%)
pT2	4 (3.8%)	3 (2.8%)
Benign histology	2 (1.9%)	3 (2.8%)
**Grade, *n* (%)**		
PUNLMP	2 (1.9%)	1 (0.9%)
Low grade	58 (55%)	60 (56%)
High grade	42 (40%)	42 (40%)
Undefined b	2 (1.9%)	1 (0.9%)
Benign histology	2 (1.9%)	3 (2.8%)
**EORTC risk group, *n* (%)**		
Low	42 (40%)	43 (40%)
Intermediate	26 (25%)	25 (23%)
High	27 (25%)	29 (27%)
Very high	4 (3.8%)	3 (2.8%)
Undefined	1 (0.9%)	1 (0.9%)
Benign histology	2 (1.9%)	3 (2.8%)
MIBC	4 (3.8%)	3 (2.8%)
**DM present, *n* (%)**		
Yes	53 (50%)	64 (60%)
No	40 (38%)	34 (35%)
Undefined	13 (12%)	6 (5.6%)

cTURBT = conventional transurethral resection of bladder tumour; DM = detrusor muscle; EORTC = European Organisation for Research and Treatment of Cancer; ERBT = en bloc resection of bladder tumours; MIBC = muscle-invasive bladder cancer; PUNLMP = papillary urothelial neoplasm of low malignant potential.

a Percentage of tumours successfully resected en bloc.

b Two pTa tumours with undefined grade and one pT1b tumour with pure squamous cell carcinoma.

### 3.2 Complications

Perioperative complications were observed in 33 (31%) of patients with ERBT versus 46 (41%) of patients with cTURBT, and postoperative complications in 19 (14%) versus 29 (26%) patients (Supplementary Table 1 and 2). The most frequent were catheter-related (Clavien-Dindo Grade I). Severe complications were rare.

### 3.3 Pathology

Four specimens were missing for pathology review, leaving 209 specimens (103 ERBT, 106 cTURBT) for analysis on the primary end point. T-stage remained unchanged after central review in 82 (80%) of ERBT and 86 (81%) of cTURBT specimens, corresponding to a difference of −1.5% (−12.3 to 9.3; *p* = 0.8; Table 3). Tumour grade remained unchanged in 83 (81%) vs 83 (78%), respectively.

**Table 3:** Pathology review and subsequent pathological findings

	**Randomisation**		
	**En bloc**, *n* = 106	**cTURBT**, *n* = 107	***p* Value**
**Pathology review of the initial specimen**	**En bloc**, *n* = 103 a	**cTURBT**, *n* = 106 a	
**Unchanged T-stage, *n* (%)**	82 (80%)	86 (81%)	
Difference, % points (95% CI)	−1.5 (−12 to 9.3) b	–	0.8 c
Upstaged, *n* (%)	13 (13%)	15 (14%)	
Downstaged, *n* (%)	8 (7.7%)	5 (4.7%)	
**Unchanged grade, *n* (%)**	83 (81%)	83 (78%)	
Difference, % points (95% CI)	+2.3 (−8.7 to 13) b	–	0.7 c
Upgraded, *n* (%)	11 (11%)	9 (8.5%)	
Downgraded, *n* (%)	8 (7.8%)	12 (11%)	
Undefined, n	1	2	
**Presence of fragments without DM or epithelial cells, *n* (%)**	40 (40%)	70 (67%)	
Difference, % points (95% CI)	−26 (−40 to −13) b	–	<0.001 d
Unknown	4	1	
**Percentage of thermal damage, %, median (Q1–Q3)**	5 (1–15)	15 (5–20)	
Difference, % points (95% CI)	−5.0 (−9.0 to −1.0) e	–	<0.001 f
Unknown	2	0	
**Pathology at reTURBT, *n* (%)**			>0.9
No residual tumour	18 (78%)	15 (79%)	
Residual tumour	5 (22%)	4 (21%)	
Upstaging	0	0	
**Location of residual tumour at reTURBT**			>0.9
Same as primary tumour, n	5/5	3/4	
**Pathology at cystectomy**			>0.9
No residual tumour, n	1/4	1/2	
Residual tumour, n	3/4	1/2	
Upstaging, n	0	0	

CI = confidence interval; cTURBT = conventional transurethral resection of bladder tumour; DM = detrusor muscle; ERBT = en bloc resection of bladder tumours; reTURBT = resection TURBT.

a Three ERBT specimens and one cTURBT specimen were unavailable for review.

b Risk difference (95% CI) from two-sample proportion test with cTURBT as reference.

c Fisher's exact test.

d Pearson's chi-squared test.

e Hodges-Lehmann median difference (95% CI) with cTURBT as reference.

f Wilcoxon rank-sum test.

A reTURBT was performed in 23 patients with ERBT and 19 patients with cTURBT, and residual tumour was found in five (22%) and four (21%) patients (Table 3). Five and two patients underwent cystectomy in the respective groups. No patients were upstaged at reTURBT or cystectomy.

Fragments without DM or epithelial cells were observed in 40 (40%) of ERBT and 70 (67%) of cTURBT specimens, with ERBT specimens containing these fragments 26% less frequently (95% CI, −40 to −13; *p* < 0.001). Median thermal damage was 5% for ERBT and 15% for cTURBT, corresponding to a difference of −5.0% (95% CI, −9.0 to −1.0; *p* < 0.001; Table 3).

The difference in thermal damage between groups was further investigated after adjustment for surgeon expertise and remained significant (*p* = 0.008). The difference in fragments with loss of orientation also remained significant after adjustment (*p* < 0.001; Supplementary Table 3).

During central pathology review, it became evident that ERBT specimen preparation had varied across sites. This resulted in numerous specimens from the ERBT group not appearing as ERBT specimens, even though the ERBT resection and extraction had been registered as successful. Conversely, some specimens maintained ERBT morphology despite partial segmentation. Ultimately, 63 specimens were considered consistent with ERBT morphological appearance and therefore constituted the PP ERBT cohort.

In PP analyses, T-stage concordance was equal between ERBT and cTURBT. Specimen orientation and thermal damage differed significantly between groups, also after adjusting for surgeon expertise (Supplementary Tables 4 and 5).

## 4 Discussion

In this multinational RCT, we compared ERBT with cTURBT through central pathology review. Contrary to our hypothesis, staging and grading concordance was similar between groups. However, in the ERBT group, specimens showed less thermal damage and better orientation—features central to reliable pathological assessment and accurate substaging.

A key consideration when interpreting the results is the lack of a standardised specimen-handling protocol. Inking of the resection base and perpendicular slicing were performed inconsistently, introducing heterogeneity in ERBT specimen preparation and potentially reducing detectable histopathological differences between techniques. This limitation reflects trial execution. Therefore, a PP analysis was performed. Although this inherently introduces selection bias, it allowed assessment of ERBT’s intrinsic histopathological performance.

Specimen orientation was consistently superior with ERBT, aligning with the conceptual advantage of preserving specimen architecture. Thermal damage also remained significantly less in the ERBT group after adjustment, despite the specimen-handling issues. This suggests that ERBT specimens have higher quality, even with suboptimal handling or specimen fragmentation.

Previous studies with pathology reviews of cTURBT specimens reported T-stage reassignment rates higher than [17] or similar [18], [19] to ours. No previous RCTs have conducted a pathology review of ERBT versus cTURBT specimens.

Studies focusing on the pathological outcomes of ERBT have suggested that the technique yields higher rates of DM representation and improves pathological accessibility [20]. Retrospective data indicate higher feasibility of pT1a/b substaging with ERBT than with cTURBT [21]. A recent RCT reported the DM rate and the presence of artefacts as measures of pathological quality: both were similar across the two groups [22]. In the present study, the representation of DM was also similar across groups.

Therefore, it remains uncertain whether these potential pathological advantages translate into more confident pathological diagnostics.

A reTURBT is recommended for T1 tumours or after incomplete initial resections. Only one-fifth of patients in our cohort underwent reTURBT with residual tumour in 22% of ERBT and 21% of cTURBT cases. These rates are lower than in other reTURBT studies [23], [24], but did not differ between groups. Meta-analytic evidence suggests that ERBT reduces residual tumour rates [25], and that reTURBT following ERBT may confer limited benefit [26]. These findings indicate that reTURBT may be omitted in selected cases, but the low number of reTURBTs in our study limits the ability to draw conclusions.

These potential advantages are also reflected in pathologists’ real-world experiences. In a recent survey study, 68 pathologists from 23 countries reported that ERBT specimens substantially improve spatial orientation, reduce the time required for microscopic assessment, and facilitate more reliable T1 substaging [27]. This aligns with our findings and highlights that broader adoption of ERBT could enhance both the quality and efficiency of pathological assessment.

## 5 Strengths and limitations

The strengths of this study include the multinational randomised design, the central pathology review with a predefined primary end point, and a high level of real-world generalisability.

The principal limitation of this trial was the absence of a standardised specimen-handling protocol. We performed a PP analysis to evaluate outcomes under optimal conditions, though this approach inherently introduces selection bias and should be interpreted accordingly.

The primary pathological assessments were performed by the local pathology departments at the participating centres following standard practice. Assessments may have been subject to interobserver variability. This reflects a pragmatic, real-world clinical setting, which we consider a strength in terms of external validity.

The study was open-label. Surgeons could not be blinded to randomisation. Patients were not blinded, as their knowledge of the randomisation was considered to have no impact on the primary end point. Pathologists were not aware of the randomisation but could not be blinded to specimen type.

Previous studies comparing bipolar instruments with laser found fewer bladder perforations and obturator nerve reflexes with laser. Because our objective was to compare pathological specimen quality in routine clinical practice rather than evaluate specific devices, instrument selection was left to the surgeon’s preference.

## 6 Conclusions

In this multinational randomised trial with central pathology review, T-stage concordance was similar between the ERBT and cTURBT groups. However, ERBT produced specimens with superior orientation and less thermal damage. Variability in specimen handling likely limited the ability to demonstrate potential histopathological benefits. Future studies employing standardised specimen-handling protocols are warranted to clarify the pathological and clinical impact of ERBT in the management of NMIBC.

  ***Author contributions:*** Ninna K. Nielsen had full access to all the data in the study and takes responsibility for the integrity of the data and the accuracy of the data analysis.

*Study concept and design*: Nielsen, Stifter-Vretenar, Jensen, Kingo.

*Acquisition of data*: Nielsen, Milling, Hjort, Vásquez, Haug, Sørensen, Olsen, Fabrin, Vjaters, Rudlang, Sepp, Nurminen, Geertsen, Lam, Jakobsen, Graugaard-Jensen, Stifter-Vretenar, Jensen, Kingo.

*Analysis and interpretation of data*: Nielsen, Hjort, Jakobsen, Jensen, Kingo.

*Drafting of the manuscript*: Nielsen.

*Critical revision of the manuscript for important intellectual content*: Milling, Hjort, Vásquez, Haug, Sørensen, Olsen, Fabrin, Vjaters, Rudlang, Sepp, Nurminen, Geertsen, Lam, Jakobsen, Graugaard-Jensen, Stifter-Vretenar, Jensen, Kingo.

*Statistical analysis*: Nielsen, Hjort, Jensen.

*Obtaining funding*: Nielsen, Jensen.

*Administrative, technical, or material support*: None.

*Supervision*: Lam, Jakobsen, Graugaard-Jensen, Jensen, Kingo.

*Other* (Pathology review): Stifter-Vretenar.

  **Ninna K. Nielsen:** Conceptualisation, Data curation, Formal analysis, Funding acquisition, Investigation, Methodology, Project administration, Visualisation, Writing – original draft.

**Rikke Vilsbøll Milling:** Investigation, Writing – review & editing.

**Peter B. Hjort:** Investigation, Formal analysis, Methodology, Visualisation, Writing – review & editing.

**Juan Luis Vásquez:** Investigation, Writing – review & editing.

**Erik Skaaheim Haug:** Investigation, Writing – review & editing.

**Frederikke Eichner Sørensen:** Investigation, Writing – review & editing.

**Kasper Ørding Olsen:** Investigation, Writing – review & editing.

**Knud Fabrin:** Investigation, Writing – review & editing.

**Egils Vjaters:** Investigation, Writing – review & editing.

**Trine Møller Rudlang:** Investigation, Writing – review & editing.

**Teesi Sepp:** Investigation, Writing – review & editing.

**Pertti Nurminen:** Investigation, Writing – review & editing.

**Louise Geertsen:** Investigation, Writing – review & editing.

**Gitte W. Lam:** Investigation, Supervision, Writing – review & editing.

**Jakob K. Jakobsen:** Investigation, Supervision, Writing – review & editing.

**Charlotte Graugaard-Jensen:** Investigation, Supervision, Writing – review & editing.

**Sanja Stifter-Vretenar:** Conceptualisation, Investigation, Writing – review & editing.

**Jørgen B. Jensen:** Conceptualisation, Funding acquisition, Investigation, Methodology, Supervision, Writing – review & editing.

**Pernille S. Kingo:** Conceptualisation, Investigation, Supervision, Writing – review & editing.

  ***Financial disclosures:*** Ninna K. Nielsen certify that all conflicts of interest, including specific financial interests and relationships and affiliations relevant to the subject matter or materials discussed in the manuscript (eg, employment/ affiliation, grants or funding, consultancies, honoraria, stock ownership or options, expert testimony, royalties, or patents filed, received, or pending), are the following:

Juan Luis Vásquez reports advisory board roles for MSD, Lina Medical, AMBU, and Photocure; speaker roles for Olympus, AMBU, and Medac; and interest in a patent for an electrode assembly for better electric field distribution.

Jakob Kristian Jakobsen reports a research grant from the Novo Nordisk Foundation; travel support and a sponsored research agreement with Medac; and consultancy work for Cystotech.

Jørgen Bjerggaard Jensen reports advisory board membership for Ferring, Roche, Cepheid, Urotech, Olympus, AMBU, Janssen, and Cystotech; speaker roles for Medac, Olympus, Photocure ASA, and Conmed; and research collaborations with Medac, Photocure ASA, Roche, Ferring, Olympus, Intuitive Surgery, Astellas, Cepheid, Nucleix, Urotech, Pfizer, AstraZeneca, MeqNordic, Laborie, OneMed, AMBU, and Cystotech.

The remaining authors have nothing to disclose.

  ***Funding/Support and role of the sponsor:*** This study was completed as part of a PhD funded by Novo Nordisk Fonden Project Grant in Surgical Research (ref. NNF19OC0058534) and Nordic Cancer Union Research Grant (ref. A16003).

The funding organisations had no role in the study design, conduct, data analysis, or interpretation.

  ***Declaration of generative AI and AI-assisted technologies in the manuscript preparation process:*** During the preparation of this work, the author used OpenAI ChatGPT-5 to improve readability and language. After using this tool/service, all authors reviewed and edited the content as needed and take full responsibility for the content of the published article.

## Appendix A Supplementary material

Supplementary Data 1 Supplementary material consists of tables containing information on peri- and postoperative complications, adjusted analyses, and per-protocol analyses.
