# Supplementary material for: Histopathological Advantages of En Bloc Resection in Non–muscle-invasive Bladder Cancer: A Multinational Randomised Controlled Clinical Trial
Source: Eur Urol Open Sci. 2026 Jul 2;90:69–76. doi: 10.1016/j.euros.2026.06.005 (PMC13351398; doi:10.1016/j.euros.2026.06.005)
Supplement: Supplementary Data 1 — Supplementary material consists of tables containing information on peri- and postoperative complications, adjusted analyses, and per-protocol analyses. [file mmc1.docx]

**Supplementary tables**

|  | **Randomisation** | |
| --- | --- | --- |
|  | **En bloc**, n = 106 | **cTURBT**, n = 107 |
| **None, n (%)** | 73 (69%) | 61 (57%) |
| **Clavien Dindo Grade I, n (%)^*^** |  |  |
| **Bladder perforation** (managed conservatively) | 11 (10%) | 13 (12%) |
| I**ndwelling catheter placement** (beyond routine management) | 18 (17%) | 17 (16%) |
| Bleeding from the urethra | 10 (9.4%) | 8 (7.5%) |
| Deep resection | 2 (1.9%) | 5 (4.7%) |
| Large resection area | 4 (3.8%) | 2 (1.9%) |
| Meatus dilatation/urethrotomy in same procedure | 1 (0.9%) | 2 (1.9%) |
| Postoperative urinary retention | 1 (0.9%) | 0 (0%) |
| **JJ-stent placement** (same procedure) | 0 (0%) | 4 (3.7%) |
| **Hospital admission** (beyond routine management) | 2 (1.9%) | 2 (1.9%) |
| **Supplementary Table 1. Perioperative complications classified according to the Clavien-Dindo system.  ^*^**Percentages represent patients experiencing the listed complication; patients could experience more than one complication. | | |

|  | **Randomisation** | |
| --- | --- | --- |
|  | **En bloc**, n = 106 | **cTURB**, n = 107 |
| **None** | 91 (86%) | 79 (74%) |
| **Clavien Dindo Grade I, n (%)^*^** | 11 (9.4%) | 24 (22%) |
| Urinary retention or bleeding requiring indwelling catheter placement | 8 | 17 |
| Catheter clotting requiring re-catheterisation | 2 | 4 |
| Lower urinary tract infection requiring oral antibiotics | 2 | 6 |
| **Clavien Dindo Grade II, n (%)^*^** | 3 (2.8%) | 2 (1.9%) |
| Urinary tract infection with fever requiring intravenous antibiotics | 2 | 1 |
| Pulmonary infection requiring antibiotic treatment | 1 | 1 |
| **Clavien Dindo Grade IIIb, n (%)^*^** | 2 (1.9%) | 5 (4.7%) |
| Active bleeding requiring surgical intervention within 48 hours | 1 | 5 |
| Renal pelvic rupture (managed with JJ stent) | 1 | 0 |
| **Supplementary Table 2. Postoperative complications classified according to the Clavien-Dindo system.  ^*^**Percentages represent patients experiencing the listed complication; patients could experience more than one complication. | | |

|  | **Randomisation** | | **Unadjusted** | **Adjusted for surgeon expertise** |
| --- | --- | --- | --- | --- |
|  | **En bloc**, n = 98^*^ | **cTURBT**, n = 102^*^ | **p-value** | **p-value** |
| **Presence of fragments without DM or epithelial cells, n (%)** | 36 (38%) | 67 (66%) |  |  |
| Risk difference, % points (95% CI) | -31.9  (-45.9, -17.9) | - | <0.001*^1^* | <0.001^3^ |
| **Percentage of thermal damage, median (Q1, Q3)** | 5 (1, 15) | 15 (5, 20) |  |  |
| Risk difference, % points (95% CI) | -4.9  (-8.5, -1.3) | - | <0.001*^2^* | 0.008^4^ |
| **Supplementary Table 3. Pathology review analysis, adjusted for surgeon expertise.**  ^*^Number of complete cases (surgeon expertise available) *^1^*Pearson's chi-squared test; *^2^*Wilcoxon rank-sum test. ^3^Logistic regression, *^4^*ANCOVA | | | | |

|  | **Specimen type** | | **Unadjusted** | **Adjusted for surgeon expertise** |  |
| --- | --- | --- | --- | --- | --- |
|  | **En bloc**, n = 65^*^ | **cTURBT/Piecemeal**, n = 135^*^ | **p-value** | **p-value** |  |
| **Presence of fragments without DM or epithelial cells, n (%)** | 5 (8.1%) | 98 (74%) | <0.001*^1^* | <0.001*^3^* |  |
| **Percentage of thermal damage, median (Q1, Q3)** | 2 (0, 5) | 15 (10, 20) | <0.001*^2^* | <0.001*^4^* |  |
| **Supplementary Table 4. Per-protocol pathology review analysis, unadjusted and adjusted for surgeon expertise.** ^*^Number of complete cases (surgeon expertise available) *^1^*Pearson's chi-squared test; *^2^*Wilcoxon rank-sum test. *^3^*Logistic regression; *^4^*ANCOVA | | | | | |

|  | **Specimen type** | | **p-value***^1^* |
| --- | --- | --- | --- |
|  | **En bloc**, n = 69 | **cTURBT/Piecemeal**, n = 140 |  |
| **Unchanged T-stage, n (%)** | 60 (87%) | 108 (77%) | 0.093 |
| **Unchanged Grade, n (%)** | 60 (87%) | 106 (76%) | 0.059 |
| **Supplementary Table 5. Per-protocol pathology review analysis on primary endpoint.** *^1^*Pearson's chi-squared test | | | |
